# Carbocisteine Reduces Airway Mucus Obstruction and Alters Inflammatory Cell Populations in a Model of Muco‐Obstructive Lung Disease

**DOI:** 10.1111/imcb.70156

**Published:** 2026-08-13

**Authors:** Peter Ferris, Ryan Brown, Michael McKelvey, Daniel F. McAuley, Marcus A. Mall, Bronwen Connolly, Clifford C. Taggart

**Affiliations:** ^1^ Airway Innate Immunity Group (AiiR), School of Medicine, Dentistry and Biomedical Sciences, Wellcome‐Wolfson Institute for Experimental Medicine Queen's University Belfast Belfast UK; ^2^ School of Medicine, Dentistry and Biomedical Sciences, Wellcome‐Wolfson Institute for Experimental Medicine Queen's University Belfast Belfast UK; ^3^ Department of Pediatric Respiratory Medicine, Immunology and Critical Care Medicine Charité – Universitätsmedizin Berlin, Corporate Member of Freie Universität Berlin and Humboldt‐Universität Berlin Germany; ^4^ German Center for Lung Research (DZL), Associated Partner Site Berlin Berlin Germany; ^5^ German Center for Child and Adolescent Health (DZKJ), Partner Site Berlin Berlin Germany

**Keywords:** airway inflammation, carbocisteine, muco‐obstructive lung disease, mucus clearance

## Abstract

Muco‐obstructive lung diseases, including chronic obstructive pulmonary disease (COPD), cystic fibrosis (CF), and bronchiectasis, are characterized by excessive mucus production, airway surface dehydration, impaired mucociliary clearance, and progressive lung function decline. As the efficacy of current therapies often declines with muco‐obstructive disease progression, contributing significantly to morbidity and mortality, there is a need for improved treatments that address underlying defects in mucus homeostasis and airway physiology. In this study, we evaluate the effects of carbocisteine, a mucoactive therapeutic, in the βENaC‐transgenic (βENaC‐Tg) model of muco‐obstructive lung disease. Short‐term carbocisteine administration significantly reduced airway mucus obstruction, potentially through decreased concentrations of Muc5ac and Muc5b. Notably, carbocisteine treatment modulated key inducers of mucus production, such as interleukin (IL)‐13 and the upstream promoter cytokine IL‐17, suggesting broader effects on mucoregulatory pathways. Carbocisteine administration was observed to alter mononuclear cell populations, impacting specific inflammatory subsets of CD11b alveolar macrophages and Ly6c monocytes, indicating immunomodulatory effects, either directly or secondary to improved mucus clearance. However, despite changes in immune cell populations, short‐term administration failed to mitigate lung damage and inflammation associated with established muco‐obstructive lung disease. These findings demonstrate the potent mucoactive effect of carbocisteine in established muco‐obstructive lung disease, through a broader mechanism of action than previously understood, with the potential to modulate inflammatory responses for preventive or long‐term treatment strategies.

## Graphical abstract

Mice with a muco‐obstructive lung disease phenotype were treated with the mucoactive drug, carbocisteine, which resulted in reduced airway mucus and an alteration in the lung mononuclear cell population which was either as a direct effect of, or secondary to, improved mucus clearance.

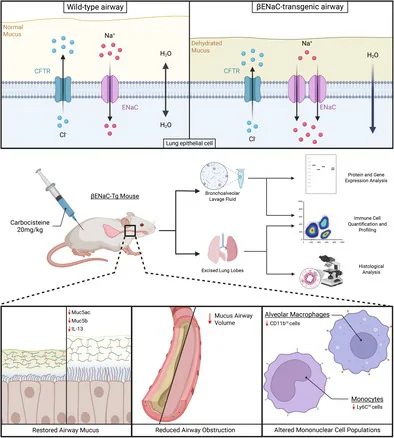

## 1 Introduction

Muco‐obstructive lung diseases, including chronic obstructive pulmonary disease (COPD), cystic fibrosis (CF), and bronchiectasis, represent a significant healthcare burden worldwide. These conditions are characterized by excessive mucus production and dehydration, airway obstruction, impaired mucociliary clearance, and declining pulmonary function [1, 2]. A primary feature of disease progression is the imbalance between proteases and anti‐proteases in the airways, contributing to extracellular matrix degradation, chronic inflammation, and progressive lung damage [3]. Despite affecting millions globally and contributing substantially to morbidity and mortality, current treatments are largely supportive and aimed at symptom management rather than addressing underlying disease mechanisms [4, 5]. However, as the efficacy of existing therapies tends to decline with disease progression, there is an urgent need for improved treatment strategies and approaches targeting the fundamental defects in mucus homeostasis and airway function.

Mucoactive therapeutics are routinely employed as long‐term treatment strategies to manage symptoms and delay disease progression of muco‐obstructive disorders [6, 7]. Distinct from alternative mucoactive agents, carbocisteine is a conventionally prescribed therapeutic, which indirectly modulates mucus production and viscosity, restoring the viscoelastic properties of airway secretions to improve clearance [8]. In addition to the principal function of modulating mucus viscosity, carbocisteine has been shown to exert anti‐inflammatory effects through free‐radical scavenging and pleiotropic mechanisms, demonstrating the capacity of carbocisteine to mitigate inflammation through suppression of NF‐κB and ERK1/2 MAPK phosphorylation [9, 10, 11, 12]. However, data reported from various clinical studies debate the efficacy and potency of carbocisteine treatment in muco‐obstructive lung disease, demonstrated to mitigate exacerbations in patients with COPD and chronic bronchiectasis, while no discernible benefit has been observed in individuals with CF [13, 14, 15]. Furthermore, an understanding of the mechanism of action of carbocisteine treatment effectiveness derived from in vivo models of mucus obstruction remains limited.

In this study, we aimed to evaluate the therapeutic impact of carbocisteine on mucus regulation, airway inflammation, and disease progression in a model of muco‐obstructive lung disease, where airway‐specific upregulation of the β subunit of the epithelial sodium channel (ENaC) leads to airway dehydration, mucus plugging, inflammation, and lung damage [16, 17].

## 2 Methods

### 2.1 Experimental Animals

All mice were managed and treated in strict adherence to the Animals (Scientific Procedures) Act 1986, and the guidelines established by the Queen's University Belfast Ethical Review Committee. Throughout the duration of study, mice were provided unrestricted access to food and water, while maintained under a standard 12 h light–dark cycle. Experiments were carried out at the same time of day across all experimental groups within a study to reduce variability associated with circadian rhythms. βENaC‐Tg mice on a C57BL/6 background as previously described [18]. At the age of 5 weeks (35 days), βENaC‐Tg and WT mice were assigned to experimental groups using a blinded randomization process, with an equal proportion of male and female mice allocated to both control and treatment groups. Mice were weighed daily to receive either 20 mg/kg carbocisteine (Sigma‐Aldrich, C0470000) or an equivalent volume of vehicle (0.9% sterile saline solution w/1% carboxymethylcellulose) alone as control. Treatments were administered intraperitoneally twice‐daily for 5 consecutive days. The blinding was maintained throughout the experimental procedures and data analysis.

### 2.2 Bronchoalveolar Lavage Fluid and Tissue Collection

Mice were culled by a lethal overdose of sodium pentobarbital (≥ 100 mg/kg) via intraperitoneal injection. Lung lobes designated for histological analysis were ligated, and the lungs were double‐flushed with a weight‐adjusted volume of Gibco PBS (Thermo Fisher Scientific) three times, then stored at −80°C. The remaining ligated tissue was harvested and immediately snap‐frozen in 2‐mL tubes using liquid nitrogen, then stored at −80°C until further use. Prior to analysis, tissue was homogenized using a Stuart handheld homogenizer (Cole‐Parmer) in 2 mL (10 mL/g tissue) RIPA buffer, supplemented with Halt Protease and Phosphatase Inhibitor Cocktail (Thermo Fisher Scientific), and incubated on ice for 30 min. Samples were centrifuged at 17 000 × g for 10 min, and the resulting supernatants were collected and stored at −80°C.

### 2.3 Cell Counts and Flow Cytometry

Total cell counts were conducted utilizing a Bright Line hemocytometer (Hausser Scientific, Horsham, US) and a DMi1 light microscope (Leica Microsystems, Milton Keynes, UK) set at x400 magnification. Cell suspensions were diluted with 0.4% trypan blue solution (Sigma‐Aldrich) to identify live cells were enumerated on the four corner grids, and the resulting average count was multiplied by the respective dilution factor and by 10^4^ to obtain the number of cells/mL. To adjust for the 100 μL volume of Gibco PBS (Thermo Fisher Scientific) used for resuspending the cell pellet instead of 1 mL, the derived value was divided by 10. Subsequently, this adjusted value was divided by the volume of BAL fluid collected to determine the total cell number/mL of BAL fluid.

Whole‐lung tissue was processed by vascularly perfusing the lungs via the right ventricle of the heart with Gibco PBS (Thermo Fisher Scientific), and once excised, the tissue was minced into pieces with scissors, digested with 0.2% collagenase type II (Worthington Biochemical, Berkshire, UK) and 20 μg/mL deoxyribonuclease I at 37°C with agitation for 30 min, then strained through a 100 μm mesh (Sigma‐Aldrich). Alternatively, BAL fluid was obtained by double‐flushing the lungs with a total of 1 mL of Gibco PBS, which was then strained through a 100 μm mesh (Sigma‐Aldrich). Samples were centrifuged (500 × g for 10 min at 4°C), and supernatant was discarded; cells were then incubated in 1 mL of red blood cell lysis buffer (BD Biosciences) for 5 min at room temperature. Following incubation, the samples were washed and resuspended in DMEM supplemented with 10% FBS (Thermo Fisher Scientific). Total cell numbers and viability were assessed using the trypan blue exclusion and up to 1 × 10^6^ cells were transferred to a Nunc 96‐Well conical bottom microwell plate (Thermo Fisher Scientific). Cells were incubated with FcBlock (BD Biosciences) in FACS buffer (Gibco PBS supplemented with 1% FBS (Thermo Fisher Scientific) and 0.5% 5 mM EDTA (Sigma‐Aldrich)) for 5 min at room temperature. To facilitate staining for specific surface markers, an antibody mastermix was added to the cell suspension and allowed to incubate for an additional 30 min at 4°C in the dark. After incubation, cells were washed and stained with fixable viability dye eFluor 780 (eBioscience) and incubated for 25 min at 4°C in the dark. Cells were filtered through a 40 μm mesh (Sigma‐Aldrich) and washed in FACS buffer before flow cytometry analysis was performed using a BD FACSCanto II flow cytometer (BD Biosciences).

### 2.4 Western Blot

For whole‐lung lysates, protein concentrations were quantified using the Bradford assay and then adjusted to an equal quantity of protein and separated on 12% SDS‐PAGE gels before being transferred onto nitrocellulose membranes (GE Healthcare, Buckinghamshire, UK). Membranes were blocked and incubated overnight at 4°C with TIMP‐1 antibody (Bio‐techne, AF980, 1:2000) or SLPI antibody (R&D Systems, AF1735, 1:2000). Additional antibodies were used as follows: GAPDH (Cell Signaling Technologies, 2118, 1:2000), IL‐17 (Novus Biologicals, NBP1‐76337, 1:1000), IL‐33 (R&D Systems, AF3626, 1:500), MMP‐2 (R&D Systems, AF1488, 1:1000), MMP‐9 (Novus Biologicals, NBP1‐57940, 1:800), SLPI (R&D Systems, AF1735, 1:2000), TIMP‐1 (Bio‐techne, AF980, 1:2000) and TIMP‐2 (R&D Systems, AF971, 1:1000). BAL fluid samples were separated on 1.2% agarose gels and transferred to nitrocellulose membranes (GE Healthcare, Buckinghamshire, UK) via a vacuum blot transfer system (MP Biomedicals, Harrogate, UK). Membranes were blocked and incubated overnight at 4°C with Muc5ac (Santa Cruz Biotechnology, sc‐398985, 1:1000) or Muc5b (Santa Cruz Biotechnology, sc‐393952, 1:1000) antibody. After washing, membranes were incubated with the respective HRP‐conjugated secondary antibodies, developed using a chemiluminescent substrate (Western Lightning, PerkinElmer, Coventry, UK), and imaged using a Syngene G:Box with GeneSnap software (Syngene, Cambridge, UK).

### 2.5 Lung Histology and Airway Morphology

Histological and morphometric analyses were performed on lung tissue sections cut at 6 μm and stained with Alcian blue‐periodic acid Schiff (AB‐PAS) (Alfa Aesar, Ward Hill, MA) to assess airway mucus content, or hematoxylin and eosin (H&E) (Leica Biosystems Ltd., Newcastle, UK) to evaluate the destructive index (DI). Airway mucus volume was assessed using AB‐PAS‐stained lung section images from the left proximal main axial airways. Mucus plugging was quantified by measuring the cross‐sectional area of the main axial airway and the luminal area containing positively stained mucus, using the ImageJ 1.54v software (NIH) and Microsoft Excel 2023. The DI was used to quantify alveolar destruction [19]. Using ImageJ 1.54v software (NIH), a 42‐point grid was overlaid onto the H&E images, with each point corresponding to specific structures categorized as either “normal” or “destroyed” alveolar and duct spaces. Alveolar and duct spaces were considered “normal” if their walls were intact or disrupted in only one location, while they were classified as “destroyed” if their walls showed two or more breaks or exhibited notable lesions, such as abnormal epithelial cells or isolated islands of lung parenchyma within duct lumens. The percentage of alveolar destruction was calculated based on these classifications and averaged using Microsoft Excel 2023 to derive a DI value for each mouse. Additionally, a measure of distal airway enlargement, the mean linear intercept (MLI), was calculated by overlaying five horizontal lines onto the H&E images using the ImageJ 1.54v software (NIH) [20]. The number of alveolar walls intersecting the grid formed by these five lines was quantified and averaged across 10 images per mouse, yielding an overall average of intercepts. This average was then divided by the length of the grid line using Microsoft Excel 2023 to determine the mean airspace diameter (chord length).

### 2.6 ELISA

ELISAs were performed according to the manufacturers' protocols using 96‐well Nunc Maxisorp microplates (Thermo Fisher Scientific). Wells were coated with 100 μL of capture antibody for IL‐13 (R&D Systems, M1300CB), KC (R&D Systems, DY453) or Osteopontin (R&D Systems, DY441) diluted in PBS and incubated overnight at room temperature. Plates were washed with PBST, blocked with 200 μL of 1% BSA in PBS for 1 h, and washed again. Samples and standards were diluted in reagent diluent, with standards prepared by twofold serial dilution. 100 μL of samples and standards were added to wells in duplicate and incubated for 2 h. After washing, biotinylated detection antibody was added, followed by incubation for 2 h. After washing, streptavidin–HRP was added and incubated for 20 min. TMB substrate was then added and incubated for up to 20 min. The reaction was stopped with 50 μL of 2 N sulfuric acid, and absorbance was measured at 450 nm and 570 nm (wavelength correction) using a BioTek Synergy HT microplate reader. Optical density values were corrected for background absorbance, and a standard curve was generated for concentration interpolation using GraphPad Prism 8.1.1.

### 2.7 Statistical Analysis

These data were managed using Microsoft Excel 2016, while statistical analyses were performed using GraphPad Prism 8.1.1 (GraphPad Software Inc). Data were graphically represented as scatter plots to visualize the distribution of the data, with means reported as mean ± standard error of the mean (SEM). Prior Before statistical analysis, normality tests were performed to determine whether to apply parametric (e.g., Bonferroni's or Tukey's post hoc tests following one‐way ANOVA) or nonparametric methods (e.g., Dunn's test following Kruskal–Wallis ANOVA), as appropriate. Group comparisons were analyzed by one‐way ANOVA or Kruskal–Wallis ANOVA, depending on data distribution. Data were considered significant if a *p* value of < 0.05 was achieved. Statistical significance was indicated as follows: *p < 0.05, **p < 0.01, ***p < 0.001, ****p < 0.0001.

## 3 Results

### 3.1 Carbocisteine Administration Reduces Airway Mucus Volume and Obstruction in βENaC‐Tg Mice

**FIGURE 1:**
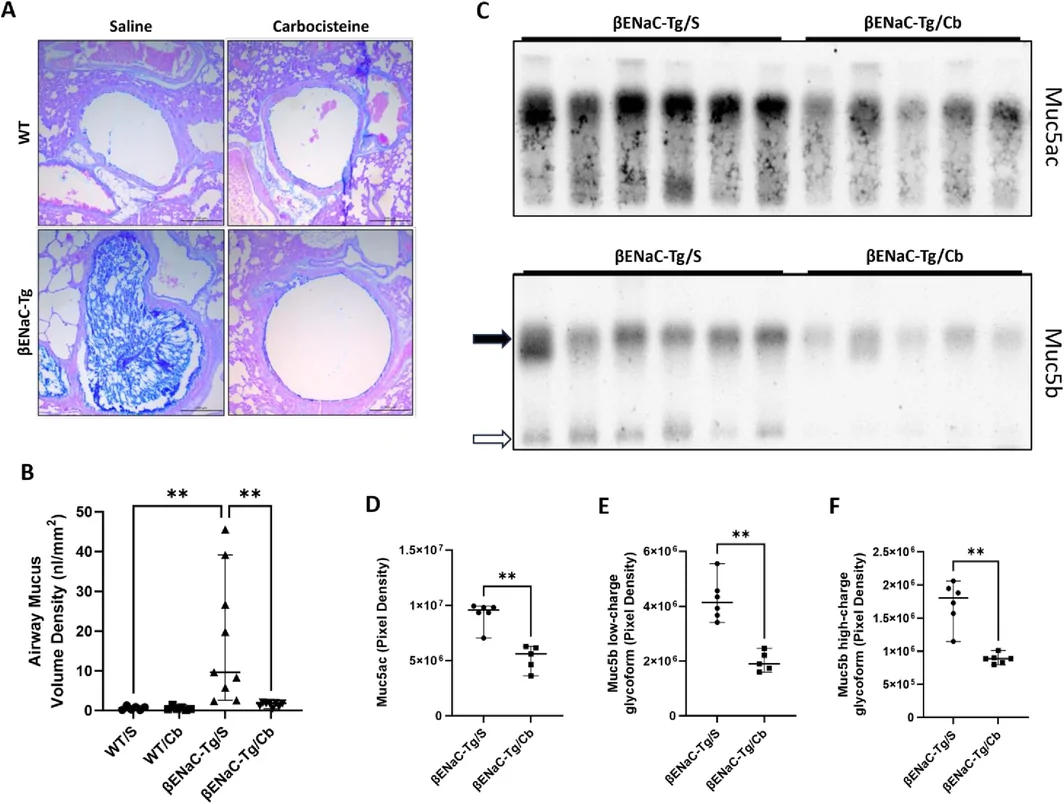
(A) Representative images (Scale bar = 200 μm) of AB/PAS‐stained lung sections from the proximal main axial airway in WT and βENaC‐Tg mice treated with either vehicle or carbocisteine. (B) Airway mucus volume density was calculated from stained sections using ImageJ (*n* = 6–9 per group). Agarose gel Western blots of (C) Muc5ac and Muc5b from vehicle or carbocisteine treated WT and βENaC‐Tg mice BALF, with solid and hollow arrows indicating the locations of the low‐charge and high‐charge glycoforms of Muc5b, respectively. Pixel densitometry was used to compare the ratios of (D) Muc5ac and Muc5b, (E) low‐charge and (F) high‐charge glycoforms (*n* = 5–6 mice per group).

Histological analysis of AB‐PAS‐stained images (Figure 1A) revealed a significant elevation in airway mucus volume in βENaC‐Tg control mice compared to WT littermates, as anticipated (Figure 1B). However, systemic administration of carbocisteine significantly reduced mucus volume density in βENaC‐Tg populations when compared to control counterparts (Figure 1B). Moreover, mucins in airway secretions were assessed by Western blot (Figure 1C). Both Muc5ac (Figure 1D) and Muc5b glycoforms, including low‐charge (Figure 1E) and high‐charge (Figure 1F) variants, were significantly reduced in the BAL fluid of carbocisteine‐treated βENaC‐Tg mice compared with controls.

### 3.2 Inducers of Airway Mucin Expression Are Altered by Carbocisteine Treatment in βENaC‐Tg Mice

**FIGURE 2:**
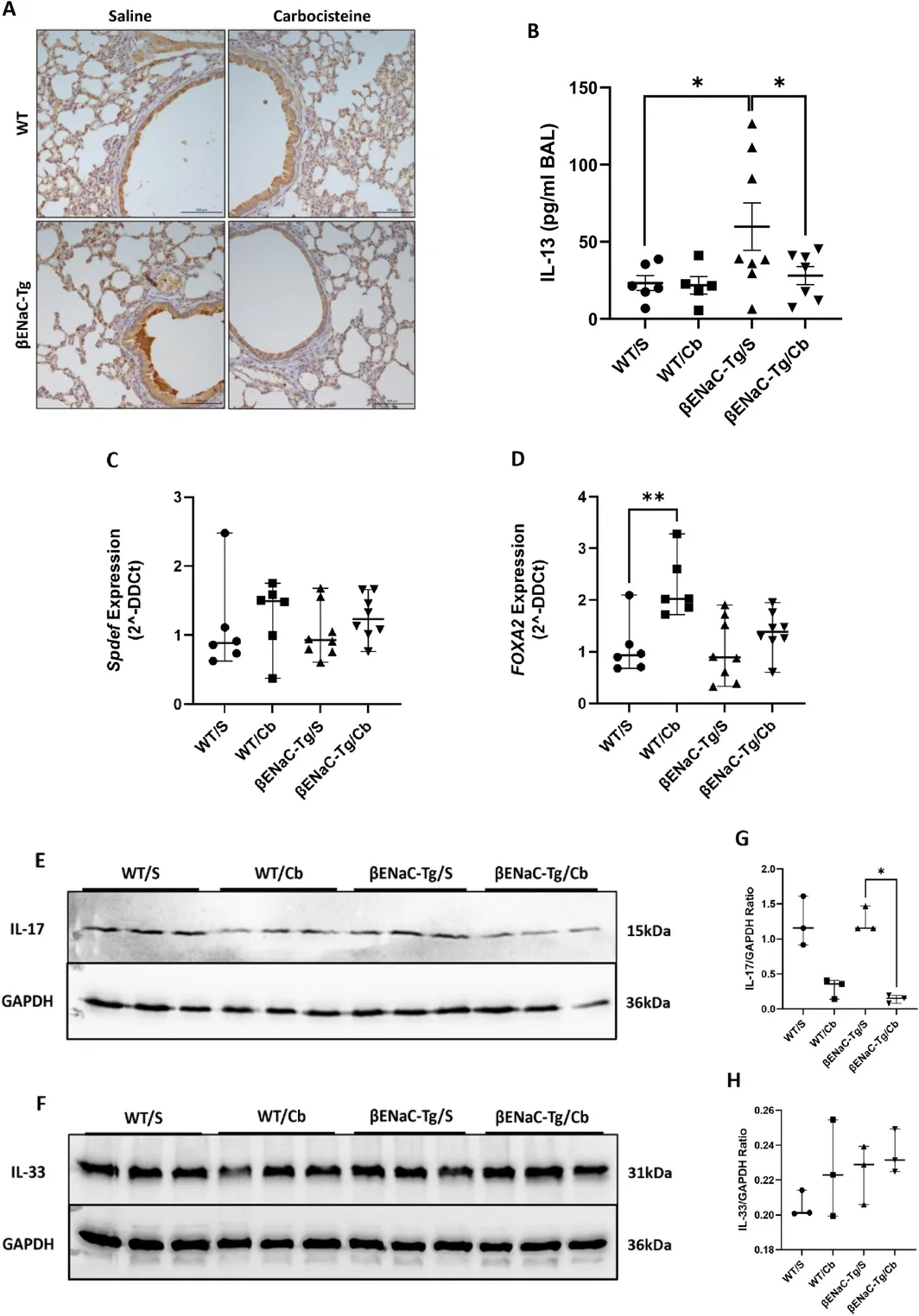
(A) Representative images (Scale bar = 100 μm) of IL‐13–stained lung sections from the proximal main axial airway and (B) IL‐13 protein concentrations in bronchoalveolar lavage fluid (BALF) quantified by Quantikine ELISA (*n* = 6–9 per group). Whole‐lung gene expression of (C) Spdef and (D) Foxa2 quantified by RT‐qPCR in WT and βENaC‐Tg mice treated with vehicle or carbocisteine (*n* = 6–9 per group). Western blot analysis of whole‐lung homogenates showing (E) IL‐17 and (F) IL‐33, with densitometric quantification of (G) IL‐17 and (H) IL‐33 normalized to GAPDH (*n* = 3 mice per group).

Histology identified intense IL‐13 staining in control βENaC‐Tg mice localized to areas of retained airway mucus, a feature absent in carbocisteine‐treated counterparts (Figure 2A). Furthermore, while IL‐13 concentrations in BAL fluid were significantly increased between WT and βENaC‐Tg control populations, systemic administration of carbocisteine significantly reduced airways IL‐13 levels in βENaC‐Tg mice compared to respective control littermates (Figure 2B). Despite this reduction in IL‐13 within the βENaC‐Tg population, carbocisteine treatment had no effect on downstream mediators and regulators of the IL‐13–activated STAT6 pathway, with expression of Spdef (Figure 2C) and FOX‐A2 (Figure 2D) remaining unchanged between treatment groups. In contrast, WT mice treated with carbocisteine exhibited increased FOX‐A2 expression compared with control littermates, an effect not observed in βENaC‐Tg mice. The observed changes in IL‐13 protein levels may instead reflect modulation of upstream inducers of IL‐13‐driven pathology. This effect appears specific to Th17‐associated responses, as carbocisteine treatment significantly reduced whole‐lung IL‐17 (Figure 2E,G) protein concentrations in βENaC‐Tg mice compared with controls, while no effect was observed on IL‐33 (Figure 2F,H) protein levels in either WT or βENaC‐Tg populations.

### 3.3 Carbocisteine Increases Airway Inflammatory Cell Counts in ENaC‐Tg Mice

**FIGURE 3:**
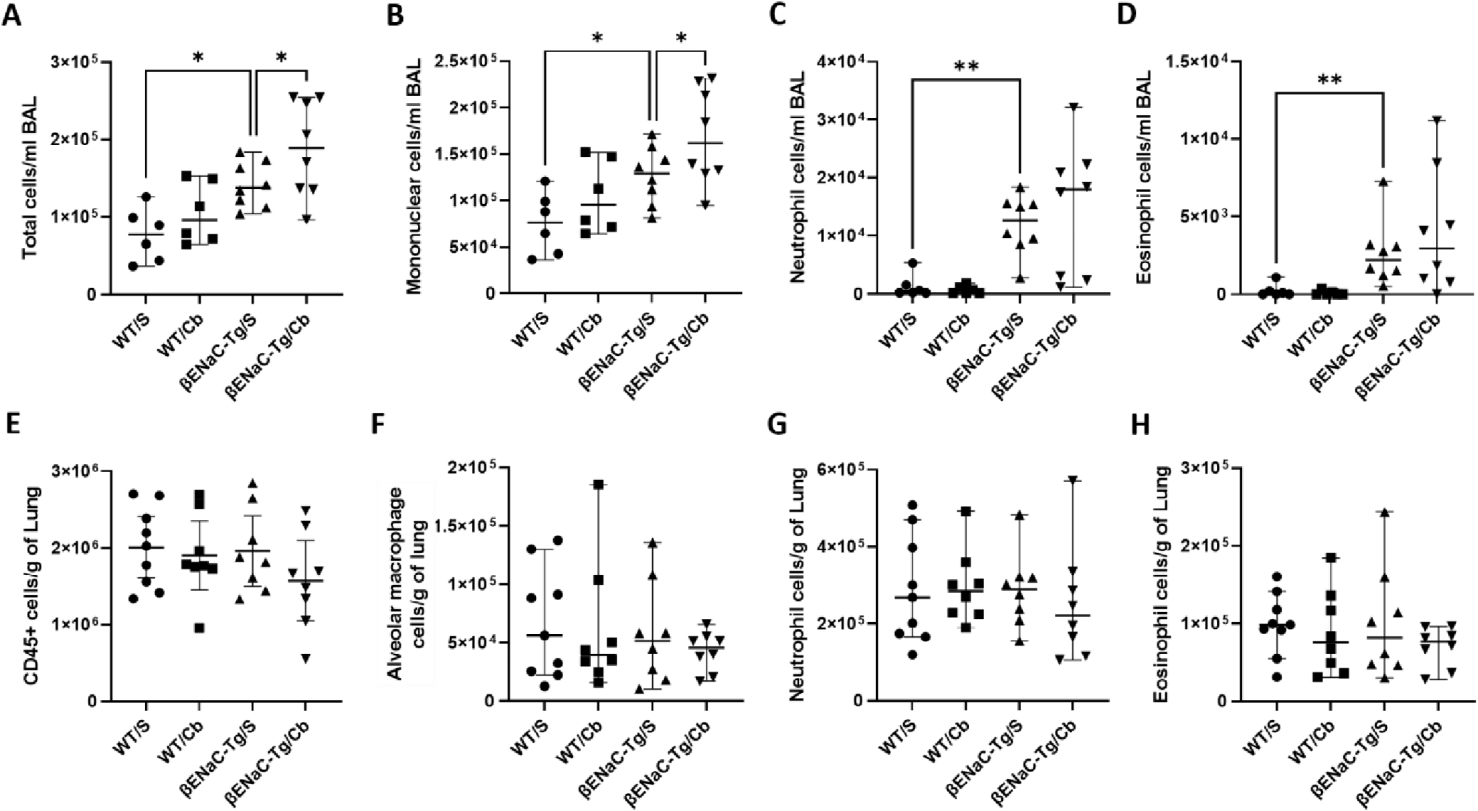
(A) Total, (B) mononuclear cells, (C) neutrophils and (D) eosinophils from vehicle or carbocisteine treated WT and βENaC‐Tg mice BALF were quantified from cytospins using ImageJ (*n* = 6–9 mice per group). (E) CD45+ cells, (F) alveolar macrophages, (G) neutrophils and (H) eosinophils from vehicle or carbocisteine treated WT and βENaC‐Tg whole‐lung homogenates were quantified via flow cytometry (*n* = 8–9 mice per group).

Persistent pulmonary inflammation represents a principal feature of muco‐obstructive lung disease, distinguished by elevated inflammatory cell infiltration into the airways, a characteristic exhibited in the βENaC‐Tg phenotype [17]. As anticipated, both total cell counts (Figure 3A) and various inflammatory cell populations (Figure 3B–D) in BAL fluid were significantly elevated in control‐treated βENaC‐Tg mice compared to WT littermates. However, carbocisteine administration significantly increased total (Figure 3A) and mononuclear cell (Figure 3B) numbers in βENaC‐Tg mice compared to their control counterparts. Despite the changes in airway inflammatory cell counts, carbocisteine administration had no discernible effect on the CD45+ (Figure 3E), alveolar macrophage (Figure 3F), neutrophil (Figure 3G), or eosinophil (Figure 3H) populations in whole‐lung tissue.

### 3.4 Carbocisteine Modulates Airway Mononuclear Cell Populations in Mice

**FIGURE 4:**
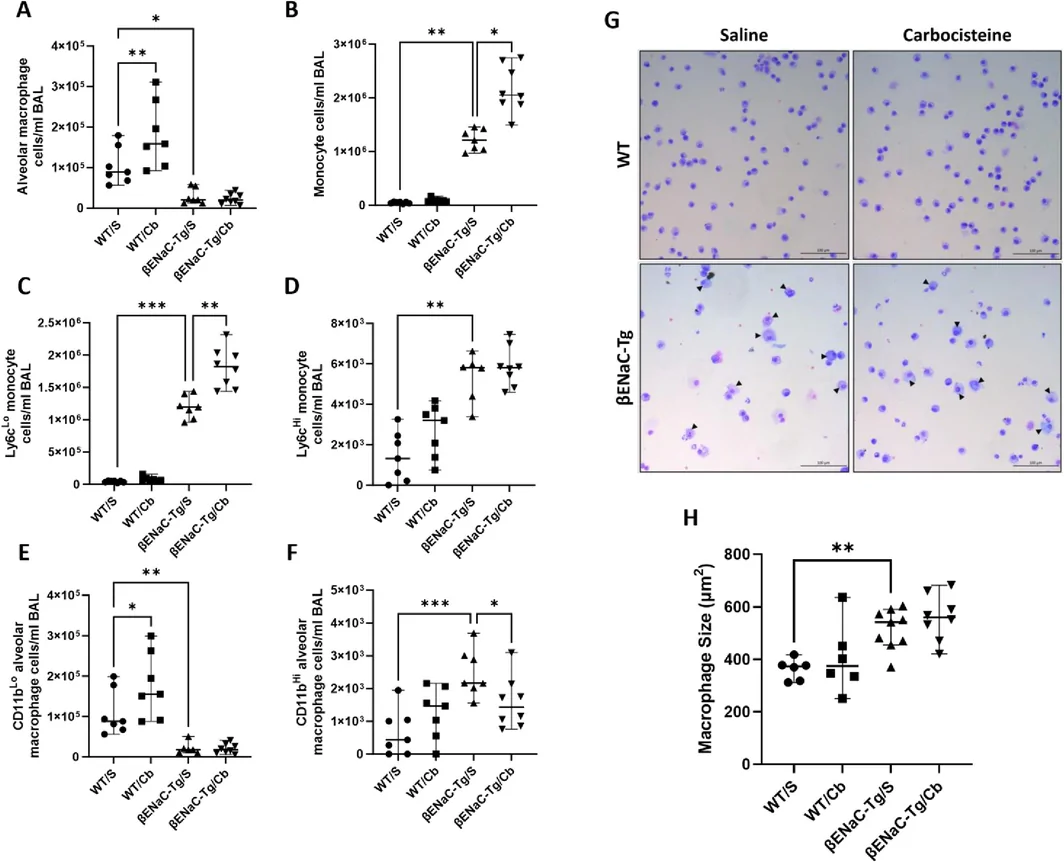
(A) Alveolar macrophage and (B) monocyte cell populations, including monocyte (C) Ly6c^Lo^ and (D) Ly6c^Hi^, and alveolar macrophage (E) CD11b^Lo^ and (F) CD11b^Hi^ subsets from vehicle or carbocisteine‐treated WT and βENaC‐Tg mice BALF were quantified via flow cytometry (*n* = 7–8 mice per group). (G) Representative images of May–GrünwaldGiemsa‐stained cells from BALF, arrows indicate morphological changes between vehicle or carbocisteine‐treated WT and βENaC‐Tg mice, with at least (H) 100 macrophages/mouse sized using ImageJ (*n* = 6–9 mice per group).

As systemic administration of carbocisteine was shown to elevate mononuclear populations localized to the airways, subsets within this population, including monocytes and alveolar macrophages, were assessed in BAL fluid. In control βENaC‐Tg mice, alveolar macrophage cell (Figure 4A) populations were significantly reduced compared to that of WT mice, where carbocisteine treatment unexpectedly elevated alveolar macrophages when compared to their control counterparts. Furthermore, carbocisteine administration significantly increased monocyte populations (Figure 4B) in βENaC‐Tg mice compared to controls, with both Ly6c^Lo^ (Figure 4C) and Ly6c^Hi^ (Figure 4D) monocyte subsets significantly increasing in control‐treated βENaC‐Tg mice compared to their WT littermates. Notably, carbocisteine treatment significantly elevated Ly6c^Lo^ (Figure 4C) monocytes in βENaC‐Tg mice compared to control‐treated counterparts, whereas no effect was observed on Ly6c^Hi^ (Figure 4D) subsets. Within alveolar macrophage populations, CD11b^Lo^ (Figure 4E) subsets were significantly reduced in control‐treated βENaC‐Tg mice, while CD11b^Hi^ (Figure 4F) cells were significantly increased compared to WT littermates. In contrast, carbocisteine treatment significantly elevated CD11b^Lo^ (Figure 4E) alveolar macrophages in WT mice, while CD11b^Hi^ (Figure 4F) subsets were significantly reduced in βENaC‐Tg mice compared to their respective control‐treated counterparts. Despite these changes in alveolar macrophage populations, cell size was significantly increased in βENaC‐Tg mice compared to WT littermates, while carbocisteine administration had no discernible impact on cell size in βENaC‐Tg mice compared to their control‐treated counterparts (Figure 6G,H).

### 3.5 Carbocisteine Treatment Has no Impact on Established Lung Damage in βENaC‐Tg Mice

**FIGURE 5:**
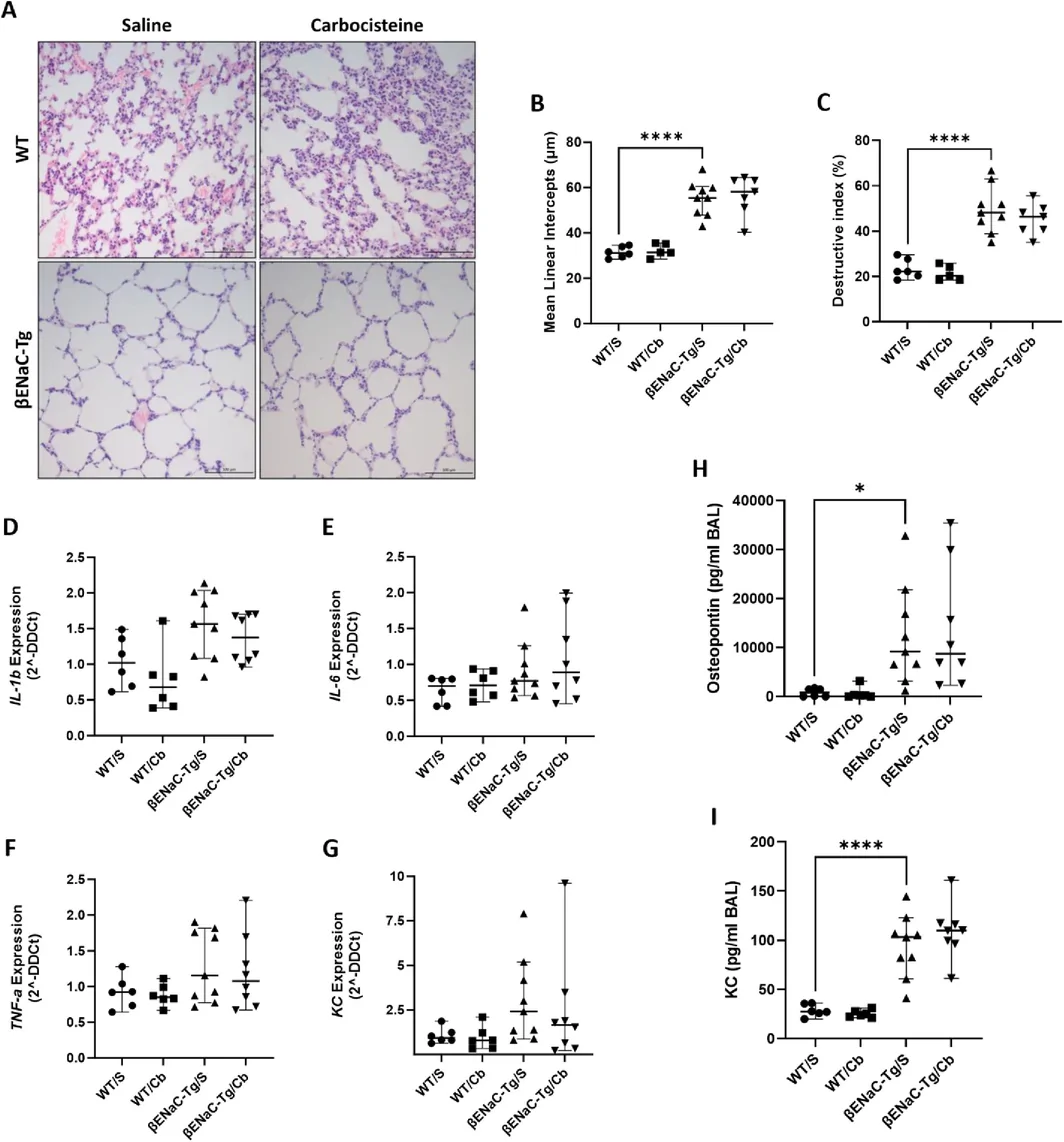
(A) Representative images of H&E‐stained lung sections from WT and βENaC‐Tg mice treated with either vehicle or carbocisteine. Histological analysis was used to quantify the (B) mean linear intercept and (C) destructive index values, which were calculated from stained sections using ImageJ (*n* = 6–9 per group). Whole‐lung gene expression of (D) IL‐1β, (E) IL‐6, (F) TNF‐α and (G) KC from WT and βENaC‐Tg mice treated with either vehicle or carbocisteine was quantified by RT‐qPCR. (H) Osteopontin and (I) KC concentrations from vehicle or carbocisteine‐treated WT and βENaC‐Tg mice BALF quantified by ELISA (*n* = 6–9 mice per group).

As distal airspace enlargement is a recognized characteristic of βENaC‐Tg lung pathology that contributes to a decline in pulmonary function in muco‐obstructive disorders, histological analysis of H&E‐stained lung sections (Figure 5A) was performed to assess the effect of treatment on alveolar destruction [16]. As expected, significant increases in MLI (Figure 5B) and DI (Figure 5C) values between the lungs of WT and βENaC‐Tg mice indicate substantial alveolar enlargement and a loss of structural integrity. No discernible effects of genotype or carbocisteine treatment were observed on the gene expression of IL‐1β (Figure 5D), IL‐6 (Figure 5E), KC (Figure 5F), or TNF‐α (Figure 5G), suggesting carbocisteine does not affect the expression of pro‐inflammatory cytokines, while treatment in this time frame was insufficient to limit the progression of lung damage. Moreover, airway levels of the pro‐inflammatory cytokines osteopontin (Figure 5E,H) and KC (Figure 5E,I) were significantly elevated in βENaC‐Tg mice compared to WT littermates, while carbocisteine treatment showed no effect when compared to control counterparts.

### 3.6 Whole‐Lung Protease and Antiprotease Protein Levels Are Altered by Systemic Carbocisteine

**FIGURE 6:**
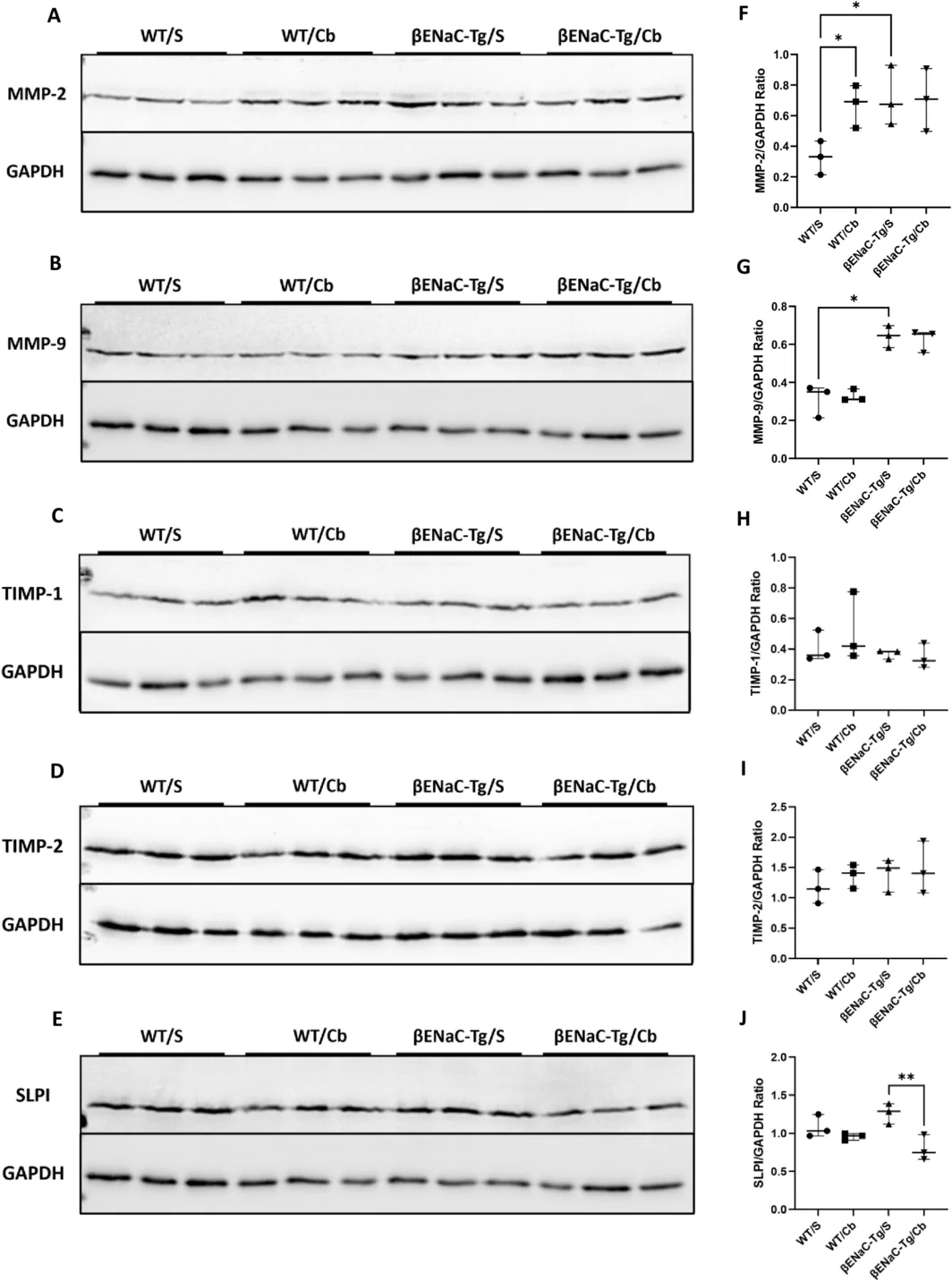
Western blots of (A) MMP‐2, (B) MMP‐9, (C) TIMP‐1, (D) TIMP‐2, and (E) SLPI from whole‐lung homogenates of WT and βENaC‐Tg mice treated with either vehicle or carbocisteine, with pixel densitometry of (F) MMP‐2, (G) MMP‐9, (H) TIMP‐1, (I) TIMP‐2, and (J) SLPI compared to ratios of GAPDH as a loading control (*n* = 3 mice per group).

Excessive protease activity is commonly observed in the pathogenesis of muco‐obstructive disorders, as airway surface dehydration increases MMP expression, and an imbalance in anti‐protease activity disrupts tissue remodeling and repair, contributing to disease progression [3]. Whole‐lung protein concentrations of MMP‐2 (Figure 6A,F) and MMP‐9 (Figure 6B,G) were significantly increased in untreated βENaC‐Tg mice compared to their WT littermates, as anticipated. However, while carbocisteine administration had no discernible impact on protease levels in the βENaC‐Tg population, treatment significantly elevated MMP‐2 (Figure 6A,F) protein levels in WT mice when compared to control counterparts. While neither genotype nor carbocisteine affected protein levels of TIMP‐1 (Figure 4C,H) and TIMP‐2 (Figure 6D,I) in whole‐lung tissue, carbocisteine treatment was observed to significantly reduce SLPI (Figure 6E,J) concentrations in βENaC‐Tg mice compared to control counterparts.

## 4 Discussion

Mucoactive therapeutics, such as carbocisteine, restore the viscoelastic properties of mucus to facilitate airway clearance in muco‐obstructive disorders, thereby mitigating disease progression by reducing exacerbations [8]. However, the efficacy and potency of carbocisteine in modulating airway secretions across various muco‐obstructive pathologies and its impact on pulmonary inflammation remain unclear. Evidence supporting its treatment effectiveness from in vivo models is also limited. This study aimed to assess the therapeutic efficacy of carbocisteine in modulating mucus secretion and obstruction, while concurrently evaluating its impact on airway inflammation and disease progression in a late‐stage model of muco‐obstructive lung disease.

While all animal models of human lung disease possess inherent limitations that can affect translational interpretation, model selection should be guided by the specific pathological features under investigation. Cigarette smoke–induced models replicate environmentally driven lung injury and aspects of COPD pathogenesis and have been widely adopted to investigate disease pathogenesis and treatment [21]. However, due to limited standardization, disease development is slow, highly variable, and dependent on exposure duration, delivery method, and species‐specific susceptibility, limiting reproducibility and sensitivity for therapeutic assessment [22, 23]. In contrast, despite the absence of bronchiectasis, the βENaC‐Tg model develops spontaneous and sustained muco‐obstructive lung disease driven by airway surface liquid dehydration, resulting in impaired mucus clearance, airway mucus obstruction, chronic pulmonary inflammation, and emphysema [16]. As the present study focused on evaluating the efficacy of a mucoactive intervention rather than modeling disease etiology, the βENaC‐Tg mouse provided a robust and reproducible platform for directly assessing the effects of carbocisteine on mucus regulation and clearance independent of the variability inherent to inhalation‐based injury models.

Depletion of the airway surface liquid layer resulted in significantly elevated mucus volume density and plugging in βENaC‐Tg mice compared to WT littermates, generating the muco‐obstructive phenotype. Systemic administration of carbocisteine significantly reduced mucus volume density in βENaC‐Tg mice, an effect attributed to decreased airway Muc5ac and Muc5b protein levels [24]. Notably, the reduction in mucin secretion includes decreases in both low‐charge and high‐charge glycoforms of Muc5b, with elevated concentrations of the low‐charge variant being associated with disease severity in both CF and COPD patients [25, 26]. In contrast, direct mucolytics such as N‐acetylcysteine (NAC) and low‐concentration (3%) hypertonic saline have shown limited effectiveness in reducing mucus hypersecretion and airway obstruction in βENaC‐Tg mice with established chronic lung disease [27, 28]. However, similar to other effective mucoactive agents evaluated in the βENaC‐Tg phenotype, such as amiloride, the restricted pharmacokinetic half‐life of carbocisteine poses challenges for maintaining therapeutic concentrations and achieving sustained mucoregulatory effects in CF [15, 29]. Therefore, further optimization of dosing regimens or the development of combination therapies may be necessary to overcome these limitations and enhance the clinical efficacy of carbocisteine in patients with CF.

In addition to its potent effect on mucus secretion, carbocisteine demonstrated a broader mechanism of action by modulating regulatory pathways involved in mucus production, including inducers and enhancers of mucins. Although increasing gene expression, carbocisteine treatment reduced airway IL‐13 protein levels, potentially contributing to the observed decrease in mucus secretion. Moreover, carbocisteine treatment was associated with reduced IL‐17 protein levels, which may serve to further limit IL‐13 signaling, as reduction consequently diminishes intracellular STAT6 pathway activation to mitigate goblet cell metaplasia, subsequently decreasing mucus secretion [30, 31]. These findings reveal a more extensive mucoregulatory mechanism associated with carbocisteine treatment than previously recognized, suggesting additional therapeutic potential in attenuating disease progression and acute exacerbations in patients with established or developing muco‐obstructive disorders.

We observed an increase in both total and mononuclear cell counts in the airways of carbocisteine‐treated βENaC‐Tg mice. This increase in airway immune cells may be partially attributed to a reduction in mucus viscosity and obstruction, as thickened secretions are known to impede the mobility of inflammatory cells in muco‐obstructive lung diseases [32]. Therefore, the reduced mucus viscosity associated with carbocisteine treatment may enhance immune cell transmigration into and through the airways. In addition, decreased viscosity of secretions could reduce adhesion to airway surfaces, facilitating efficient mucus clearance and thereby promoting the removal of immune cells [32, 33]. Within monocyte populations, Ly6C serves as an antigen commonly used to distinguish pulmonary monocyte subsets, with elevated expression associated with pro‐inflammatory responses. Notably, carbocisteine treatment specifically increased the population of Ly6C^Lo^ monocytes, a subset linked to tissue repair and anti‐inflammatory functions, suggesting a potential shift toward a more regulated immune environment in the airways [34, 35]. In contrast, carbocisteine treatment also led to a significant reduction in the number of CD11b^Hi^ alveolar macrophages in the airways. Consistent with our approach, CD11b expression has been widely used to characterize alveolar macrophage phenotype, particularly under inflammatory conditions, where profiles frequently segregate based on CD11b upregulation—a marker associated with aging and a proinflammatory M1 phenotype, with elevated IL‐6, IL‐1β, and TNF‐α [36, 37, 38]. This outcome contrasts with previous studies that reported a predominance of inflammatory alveolar macrophages in the βENaC‐Tg model [39]. This discrepancy may reflect the differential modulation of immune cell subsets by carbocisteine, which could influence the recruitment and activation of macrophages in a manner distinct from what was previously understood. These observations suggest carbocisteine treatment may modulate immune cell dynamics within the airways, potentially promoting the recruitment of anti‐inflammatory monocytes while reducing the number of activated, pro‐inflammatory macrophages.

Despite potent mucoactive effects on mucus secretion, carbocisteine administration failed to ameliorate lung damage or alveolar destruction in the muco‐obstructive phenotype. While carbocisteine effectively reduced airway obstruction, markers of airway inflammation and lung damage parameters remained unchanged, and gene expression of pro‐inflammatory mediators showed no alteration. These findings contrast with previous reports demonstrating that high‐dose, prophylactic carbocisteine can significantly reduce pulmonary inflammation and tissue damage [40, 41]. The discrepancies may be attributed to differences in treatment protocols, as our study employed a 5‐day therapeutic regimen in an established disease model, whereas prior studies used longer, preventive treatment in cigarette smoke‐induced models [40, 41]. Furthermore, evidence from the βENaC‐Tg phenotype suggests that at least 14 days of intervention are necessary to observe reparative effects, indicating that short‐term treatment may be insufficient to influence airway inflammation [42]. Collectively, our results highlight the potential of long‐term or prophylactic carbocisteine administration as a more effective strategy for attenuating disease progression and mitigating lung damage in muco‐obstructive disorders [43].

Interestingly, carbocisteine treatment significantly reduced SLPI levels in βENaC‐Tg mice compared to control‐treated counterparts, whereas genotype and treatment had no effect on TIMP‐1 and TIMP‐2 protein concentrations in whole‐lung tissue. As carbocisteine has been demonstrated to mitigate NF‐κB and ERK1/2 MAPK signaling in epithelial cells, the selective reduction in SLPI may be partially attributed to signal attenuation in epithelial tissue, where SLPI is predominantly produced and tightly regulated as an inducible protective response [10, 11, 12]. Additionally, the short half‐life and rapid secretion of SLPI render whole‐lung concentrations highly responsive to changes in epithelial production [44]. In contrast, TIMP‐1 and TIMP‐2 are preferentially expressed by immune and stromal cells and are subject to more constitutive regulation, making them less sensitive to signaling changes, while greater molecular stability and extracellular matrix association likely buffer them against acute therapeutic modulation [45, 46]. Similarly, carbocisteine treatment did not affect MMP‐9 and MMP‐2 levels, both of which are largely expressed by immune and stromal cells where carbocisteine may exert reduced signaling effects during short‐term administration [47]. Collectively, these findings suggest that systemic carbocisteine administration may modulate protease and antiprotease expression in a cell‐type‐dependent manner, preferentially affecting epithelial‐derived factors while having minimal impact on immune and stromal cell‐derived proteins during short‐term treatment.

In conclusion, carbocisteine demonstrates considerable potential as a therapeutic agent for muco‐obstructive lung diseases by reducing mucus secretion, alleviating airway obstruction, and modulating airway inflammation and immune cell dynamics. However, the lack of long‐term effects on lung damage suggests that carbocisteine may be most effective when used in conjunction with other therapies that target chronic inflammation and tissue remodeling. Further studies are needed to explore the long‐term efficacy of carbocisteine and to better understand the complex interactions between mucus clearance, inflammation, and immune cell recruitment in muco‐obstructive diseases.

## Author Contributions

**Ryan Brown:** conceptualization, methodology, investigation, validation, writing – review and editing. **Daniel F. McAuley:** methodology, resources, writing – review and editing. **Marcus A. Mall:** writing – review and editing, visualization, formal analysis. **Bronwen Connolly:** supervision, writing – review and editing. **Peter Ferris:** methodology, investigation, formal analysis, data curation, writing – review and editing, writing – original draft. **Clifford C. Taggart:** conceptualization, investigation, funding acquisition, visualization, project administration, resources, supervision, writing – review and editing. **Michael McKelvey:** methodology, formal analysis, investigation, writing – review and editing.

## Funding

We wish to acknowledge funding by the Department for the Economy Northern Ireland (PhD studentship to P.F.).

## Conflicts of Interest

The authors declare no conflicts of interest.

## Data Availability

The data that support the findings of this study are available from the corresponding author upon reasonable request.
